# Financial Exploitation in Private Home Care: Findings From a Multi-State Care Manager Survey

**DOI:** 10.1093/geroni/igaf122.3773

**Published:** 2025-12-31

**Authors:** Shadi Gholizadeh, Megan Harris, Lucas Melville

**Affiliations:** TheKey, coto de caza, California, United States; TheKey, San Diego, California, United States; TheKey, San Diego, California, United States

## Abstract

Financial exploitation is a critical yet under-recognized risk in private home care, particularly for older adults living with dementia. To better understand its scope and potential safeguards, we conducted a survey of 14 private-duty care managers across 14 states who collectively oversee approximately 700 clients per month. Respondents indicated that they occasionally encounter suspected or confirmed cases of financial exploitation in their professional roles. The most frequently described concerns included theft of valuables, misuse of credit or debit cards, and solicitation of gifts or loans. A number of care managers shared that, across their careers, they had removed caregivers from cases due to concerns about financial boundaries. Despite these observations, relatively few families proactively raised financial concerns, suggesting that many situations may go undetected or unreported. Respondents expressed openness to preventive strategies, including the use of prepaid cards, financial alert systems, and structured caregiver training on boundary-setting and red flags. Most emphasized that reinforcing safeguards at the start of care would be particularly valuable, for example through clear documentation of financial preferences and explicit plans for caregiver involvement in purchases such as groceries. Findings highlight both the potential frequency and complexity of financial exploitation in private home care, as well as opportunities for structured, proactive interventions. These results underscore the shared responsibility of agencies, caregivers, and families in protecting vulnerable clients, and they point to the need for further practice, policy, and research efforts to strengthen safeguards and promote elder justice in the growing private home care sector.

